# Inverted bell-curve-based ensemble of deep learning models for detection of COVID-19 from chest X-rays

**DOI:** 10.1007/s00521-021-06737-6

**Published:** 2022-01-05

**Authors:** Ashis Paul, Arpan Basu, Mufti Mahmud, M. Shamim Kaiser, Ram Sarkar

**Affiliations:** 1 Department of Computer Science and Engineering, Jadavpur University, Kolkata, 700032 India; 2 Department of Computer Science, Nottingham Trent University, Clifton, Nottingham NG11 8NS UK; 3 Medical Technologies Innovation Facility, Nottingham Trent University, Clifton, Nottingham NG11 8NS UK; 4 Computing and Informatics Research Centre, Nottingham Trent University, Clifton, Nottingham NG11 8NS UK; 5 Institute of Information Technology, Jahangirnagar University, Dhaka, 1342 Bangladesh

**Keywords:** COVID-19 detection, Convolutional neural network, Ensemble learning, Chest X-ray, Bell-shape function

## Abstract

Novel Coronavirus 2019 disease or COVID-19 is a viral disease caused by severe acute respiratory syndrome coronavirus 2 (SARS-CoV-2). The use of chest X-rays (CXRs) has become an important practice to assist in the diagnosis of COVID-19 as they can be used to detect the abnormalities developed in the infected patients’ lungs. With the fast spread of the disease, many researchers across the world are striving to use several deep learning-based systems to identify the COVID-19 from such CXR images. To this end, we propose an inverted bell-curve-based ensemble of deep learning models for the detection of COVID-19 from CXR images. We first use a selection of models pretrained on ImageNet dataset and use the concept of transfer learning to retrain them with CXR datasets. Then the trained models are combined with the proposed inverted bell curve weighted ensemble method, where the output of each classifier is assigned a weight, and the final prediction is done by performing a weighted average of those outputs. We evaluate the proposed method on two publicly available datasets: the COVID-19 Radiography Database and the IEEE COVID Chest X-ray Dataset. The accuracy, F1 score and the AUC ROC achieved by the proposed method are 99.66%, 99.75% and 99.99%, respectively, in the first dataset, and, 99.84%, 99.81% and 99.99%, respectively, in the other dataset. Experimental results ensure that the use of transfer learning-based models and their combination using the proposed ensemble method result in improved predictions of COVID-19 in CXRs.

## Introduction

The Novel Coronavirus 2019 disease or COVID-19 caused by severe acute respiratory syndrome coronavirus 2 (SARS-CoV-2) is spreading rapidly all over the globe. The World Health Organization (WHO) declared it as a global pandemic [1] on March 11, 2020, and as of January 2021, the virus has infected more than 105,000,000 people worldwide. Though having a lower mortality rate than its predecessors, Severe Acute Respiratory Syndrome (SARS) and Middle East Respiratory Syndrome (MERS), COVID-19 has killed more than 2,200,000 people worldwide.

The standard and the definitive way to detect COVID-19 is via Reverse Transcription Polymerase Chain Reaction (RT-PCR). However, such tests are reported to have a high false-negative rate [2] and variable sensitivity. So as an alternative diagnosis method and to determine the progress of the disease in a patient’s body, chest X-rays (CXRs) and computed tomography (CT) scans are used [3]. This is due to the fact that COVID-19 causes visible abnormalities in the lungs which are visually similar yet often distinct from viral pneumonia [4]. Though chest CT scans have high sensitivity towards pulmonary diseases, they are not portable and carry a high risk of exposing health workers and the person under investigation to the virus. The CXRs being portable are considered to be a safe alternative [5] as the person under investigation can be imaged in a more isolated environment, thereby lowering the risk of spreading the virus. Although a vaccine has been developed, it will take time to vaccinate the entire world population, especially in developing countries [6].

With the recent developments in data-driven Deep Learning (DL), various DL models like convolutional neural networks (CNNs) are being used extensively to study medical images [7]. CNNs are achieving state-of-the-art performances in classification into disease classes for diagnosis and also in segmentation of the region of interest (ROI) in medical images. This is enabled by the fact that CNNs can learn local features very accurately from a given medical image which can be a CT scan or a CXR. Combining outputs of multiple classifiers to generate the final output is a popular approach to enhance the performance of classification. The combination of ensemble algorithms works on the output scores of the individual classifiers, which may have different architectures to capture different elements of data or different input vectors generated from the same data instance [8]. Existing popular rank level or confidence score level ensemble methods like majority voting, sum-rule (soft voting) [9] focus on a linear combination of the classifiers’ outputs to generate the final prediction, lacking any consideration of the output vector quality.

In this paper, we propose a novel weighted average ensemble method to combine the confidence scores of various pretrained CNN models to achieve better performance in detecting COVID-19 from CXR images. The inverted bell curve is used to assign weights to the classifiers’ outputs. The more we move further from the centre of the bell we attain higher weight values, and thus the shape of the inverted bell is utilized to calculate the weight for an output vector. Both the classifiers’ output quality and the overall performance of the classifiers are considered, thereby providing a more justifying combination of classifier outputs. Transfer learning is used where the CNN models are first pretrained on a huge dataset to learn basic image-related features. Then they transfer the knowledge with some fine-tuning to classify CXR images to help the medical practitioners in the diagnosis of COVID-19. We highlight the benefits of the proposed inverted bell-curve-based ensemble method to improve the accuracy and robustness of these transfer learning models.

To summarize, the contributions of this work are as follows:

1. We propose an ensemble of transfer learning models to classify CXR images to detect COVID-19.
2. We propose a novel ensemble method that uses an inverted Bell curve to assign weight to the output of the classifiers and performs weighted average to obtain the final output vector.
3. The proposed approach is evaluated on COVID-19 Radiography Database [10] and IEEE COVID Chest X-ray Dataset [11] and state-of-the-art results are obtained.

The remaining paper is structured as follows: Sect. 2 provides a quick review of the past methods related to the research topic under consideration. In Sect. 3, we discuss the proposed approach. Section 4 presents the results followed by a brief discussion on the same. We end with concluding remarks outlining some future research plans in Sect. 5.

## Related work

Several methods such as transfer learning, ensembling, etc., have been proposed in the literature to improve the performance of the DL models. More recently, researchers have applied these techniques in several domains of image processing like facial expression recognition [12], image fusion [13], malware classification [14], etc. Such methods have also been used in the medical image processing domain. A recent work by Dolz et al. [15] uses CNN ensembles for infant’s brain MRI segmentation. Another work by Efaz et al. [16] uses deep CNN supported ensembles for computer-aided diagnosis of malaria. Savelli et al. [17] have also developed a similar method for small lesion detection. It is, therefore, logical that similar methods have also been applied for COVID-19 detection. We highlight a few such works below.

Gianchandani et al. [18] have also used an approach where they use transfer learning and ensembling to improve the performance of their DL models. The authors have considered the VGG16, ResNet152V2, InceptionResNetV2 and DenseNet201 models in their work which are trained using transfer learning. The authors then show that a deep neural ensembling provides better results when compared to each of the models. The work utilized two datasets for training the DL models. The first one was obtained from Kaggle and used for binary classification. The second one was collected by a team of researchers in collaboration with doctors and was used for multi-class classification. It contained 1203 CXRs equally split among the 3 classes of COVID affected, normal and pneumonia affected, respectively. The accuracy and F1 scores are reported as 96.15% and 0.961 for the binary classification task, and as 99.21% and 0.99 for the multi-class classification task, respectively.

A recent work by Ouyang et al. [19] also utilizes ensembling. The authors have developed a dual-sampling attention network to detect COVID-19 in CT scan images. To deal with the imbalance in the distribution of the infection regions in the lungs, a dual-sampling strategy was used. Two separate 3D ResNet34 models were trained using different sampling strategies, and finally, the predictions were combined using weighted average ensembling. For training and validation, a dataset consisting of 2186 CT scans from 1588 patients was used. For the testing stage, another independent dataset comprising of 2796 CT scans from 2057 patients was used. The AUC, accuracy and F1 score values are reported as 0.944, 87.5% and 82.0%, respectively, on the testing dataset.

Similarly, in [20], the authors have used ensembling and iterative pruni ng to improve the classification results of their DL models. Four publicly available CXR datasets are used in the work. One pneumonia-related dataset was used for modality-specific training before training on the COVID-19 CXRs. The idea is that training on a similar dataset of CXRs will be beneficial for the DL models. The authors report their accuracy and AUC as 99.01% and 0.9972, respectively.

Zhang et al. [21] have used two-stage transfer learning and a deep residual network framework for the classification of CXR images. The authors used pretrained ResNet34 model and fine-tuned the model on a large dataset of pneumonia CXR images. The authors then used a feature smoothing layer and a feature extraction layer and utilized them along with layers transferred from the fine-tuned ResNet34 model. A fully connected layer at the end of the network produces the final output. The authors used two datasets of CXR images, one with 5860 images which were used for the first stage of training and the other one with 739 images which was used for the later stage. The testing accuracy is reported as 91.08 % by the authors.

Jaiswal et al. [22], in their work, have utilized transfer learning in DL models to detect COVID-19 in CT scan images. The authors have used the ImageNet dataset for pretraining and the SARS-CoV-2 CT scan dataset for training the models. The authors have observed that the DenseNet201 model performs the best as compared to the VGG16, ResNet152V2 and InceptionResNetV2 models. The training, testing and validation accuracies are reported as 99.82%, 96.25% and 97.40%, respectively.

Recently, several works ([23, 24]) have also been proposed which make use of optimization algorithms along with DL for COVID-19 detection. The work by Goel et al. [25] introduced an optimized CNN termed as OptCoNet for the purpose of COVID-19 diagnosis from CXRs. The proposed CNN model consists of feature extraction components and classification components as usual. However, the hyperparameters of the CNN (like learning rate, number of epochs, etc.) have been optimized by using the Grey Wolf Optimization algorithm. A dataset comprising of 2700 CXRs collected from various public repositories was used. There were three classes in all: COVID affected, pneumonia affected and normal, with 900 X-rays belonging to the COVID affected class. The authors have reported the accuracy, sensitivity, specificity and F1 score values as 97.78%, 97.75% 96.25% and 95.25%, respectively.

Ezzat et al. [26] also use a similar approach where they have used the Gravitational Search Algorithm to choose the optimal hyperparameters for a DenseNet121 CNN model. The authors go on to show that such a method performs better than the state-of-the-art InceptionV3 model. In the work, a combination of two datasets, the Cohen dataset and the Kaggle Chest X-ray Dataset, has been used. The final dataset contained 99 COVID-19-positive X-rays and 207 COVID-19-negative X-rays which also included some other diseases like pneumonia, SARS, etc. in addition to normal X-rays. The authors have reported the accuracy and F1 score of their method as 98.38% and 98%, respectively. 

The availability of a large quantity of training data is also required for the success of DL models. However, in the emerging domains, there is often a lack of training data. Waheed et al. [27] have proposed an auxiliary classifier generative adversarial network (ACGAN)-based model termed as COVIDGAN to tackle this issue. The authors have used a dataset consisting of 1124 CXRs of which 403 are COVID-19 infected, and the rest are normal. It has been derived from 3 open-sourced datasets. The authors have shown that including the synthetic images generated by COVIDGAN in a VGG16 classifier improves the performance of the model. The accuracy, F1 score, sensitivity and specificity improve to 95%, 0.95, 90% and 97%, respectively, from 85%, 0.85%, 69% and 95%, respectively.

### Research gap

As highlighted in the previous section, ensembling-based approaches are widely used in different image classification tasks among others. They have also been used in a few methods proposed for COVID-19 detection. The most common techniques used include summation, majority voting, averaging and weighted averaging of the predictions obtained from the classifiers considered for forming the ensemble. These approaches provide a significant improvement in performance in most cases. However, an important observation is that these methods do not consider the quality of the predictions while producing the output. These techniques simply apply the corresponding operation to obtain the output. We may also choose to use some secondary classifiers [28] which can make use of some learning algorithms. This learning process is based on the optimization of some metrics like accuracy or F1 score. For example, the work by Gianchandani et al. [18] mentioned previously uses a neural network-based secondary classifier. Applying the similar technique in our experimental setup does not produce a superior result. This secondary classification stage also does not give any separate treatment based on the quality of the predictions, which may explain the previous observation.

In many cases, it has been observed that classifiers obtain high accuracy on a particular task even if the quality of the predictions is inferior. Here, we assume the accuracy to be the fraction of correctly predicted classes where each prediction is the class with the maximum probability as predicted by the model under consideration. By quality, we refer to the difference among the predicted probabilities. This is to some extent a metric to measure the confidence of the model. For example, between two classifiers with predicted class probabilities as [0.8, 0.1, 0.1] and [0.4, 0.3, 0.3], we would prefer the former classifier. In terms of the accuracy viewpoint mentioned previously, both would produce the same output. However, from the quality viewpoint, the first classifier would be preferred since it predicts the class with high confidence. The differences in probability scores of the predicted class (0.8) with the other classes (0.1 and 0.1) are very high. The second classifier is said to be unsure of the prediction since the differences in scores of the predicted class (0.4) with the other classes (0.3 and 0.3) are very low. Its output is very close to the uniform random prediction of [0.33, 0.33, 0.33]. Hence, we would consider the first classifier as the better one, since the difference between the probabilities of the predicted class and the other classes is minimal.

In the present work, we use the inverted bell curve in order to minimize this issue. The aim is to introduce some robustness in the ensembling process while improving classification accuracy at the same time.

## Proposed approach

**Fig. 1:**
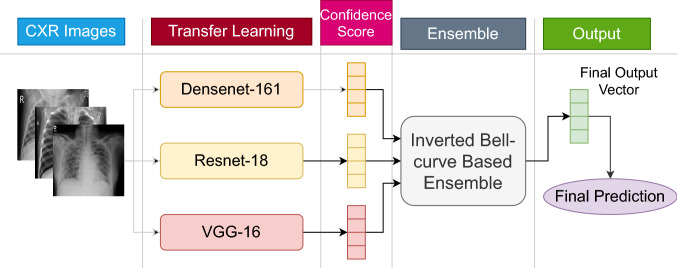
Graphical representation of the proposed ensemble method used for detecting COVID-19 from CXR images

This section describes the methods used in this study. All these methods put together to create the proposed analysis pipeline whose block diagram is shown in Fig. 1.

### Preliminaries

In the domain of computer vision, CNNs have proved to be the best tool by achieving excellence in a wide array of research problems including image classification, object detection, image segmentation, etc. The convolution layer does most of the computation. These layers convolve the input with a filter and pass it to the next layers as the output. Like the former, pooling layers do not have any weights associated with them. The pooling layers help to reduce the dimensions of the intermediate feature maps before they are passed through the activation function. Although this downsampling strategy using pooling layers loses some data, it helps in preventing overfitting and reduces the complexity of the overall network. The final convolution layer is generally followed by a fully connected network (FC), where all the neurons in one layer are connected to the outputs of the previous layer.

Activation functions are used to introduce nonlinearity in neural networks. The activation functions that we have used include: Rectified Linear Unit (ReLU), Softmax and Sigmoid. Practically, ReLU has been found to be better as compared to sigmoidal functions for intermediate activation in a network. It speeds up the convergence of stochastic gradient descent (SGD) and also reduces the vanishing gradient problem.

Dropout [29] is a method of regularization and is frequent used in CNNs. Normally, overfitting is a major problem in deep networks with a large number of parameters. Dropout randomly drops neurons along with their connections from the entire network. This prevents neurons from co-adapting too much and can be considered to be a form of model averaging but for neural networks. Batch normalization [30] is a method used to make the training of neural networks faster and more stable by reducing internal covariate shift. This is achieved by normalization of the layer inputs by recentring and scaling.

### Transfer learning

In general, CNNs require a large amount of data for their training and also for the generalizability of the trained model. On smaller datasets, there is the risk of overfitting, where the model tries to remember the training data and the corresponding output. As a result, it cannot handle input samples outside of the training dataset. This is especially relevant for the deeper and more complex models. Nowadays, CNNs are rarely trained from scratch. Transfer learning is applied where the model is first trained on a much larger dataset like ImageNet. Thereafter, the model is trained on the dataset for the task under consideration with a low learning rate. Recently, this concept has been successfully applied on various complex image processing tasks including medical image analysis. We use the same technique in this work.

Here, we consider three widely used and standard CNN models as the base learners of the proposed ensemble approach which are VGG-16 [31], ResNet-18 [32] and DenseNet-161 [33]. We choose this particular set of models as these models are able to pay attention to the different regions at an image that can produce better results with the ensemble (refer to Sect. 4.3). Along with that among all the combinations tried, the ensemble of these three models produces the best result, as shown in Table 4. We train them to obtain the confidence scores for the classes present in the dataset under consideration. The models are first pretrained on the ImageNet dataset. Then the models are trained for 20 epochs using the SGD optimization algorithm with a learning rate of 0.001.

The VGG [31] is one of the simpler and older CNN architectures first proposed by Simonyan and Zisserman. It consists of convolutional, pooling and fully connected layers. The last layer has a softmax activation and produces a 1D tensor with a dimension equal to the number of classes.

**Fig. 2:**
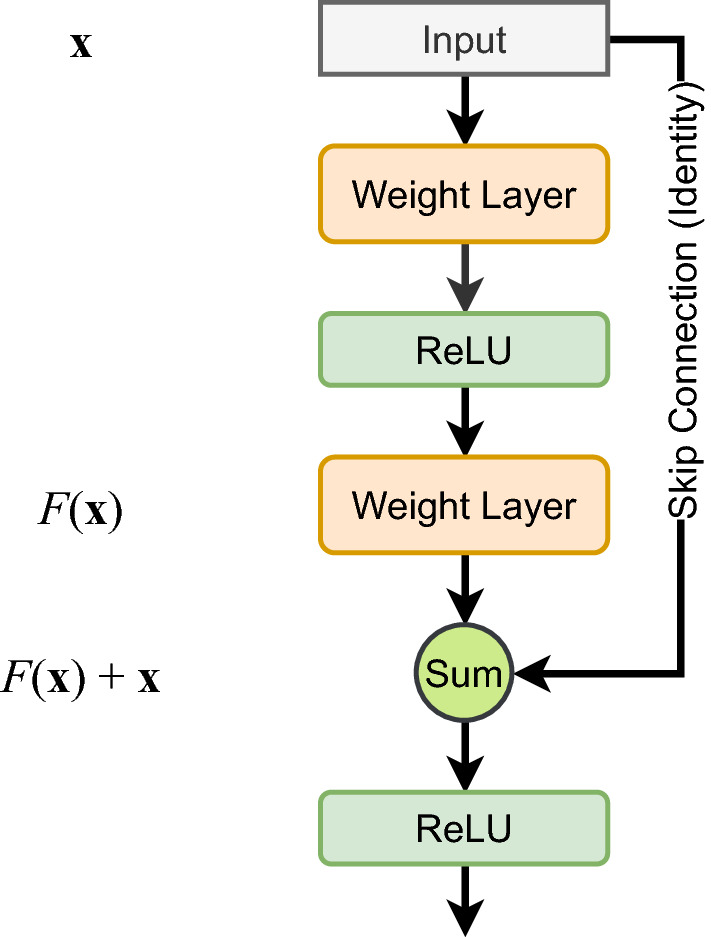
A pictorial representation of the skip connections in the ResNet architecture. Modified from [32]

The ResNet [32] architecture was first proposed to deal with the vanishing gradients problem that occurs when training very deep networks. It consists of skip connections in-between consecutive layers which reduce this problem to a great extent. This is highlighted in Fig. 2. The identity path provides an alternative route for the gradients to flow through.

**Fig. 3:**
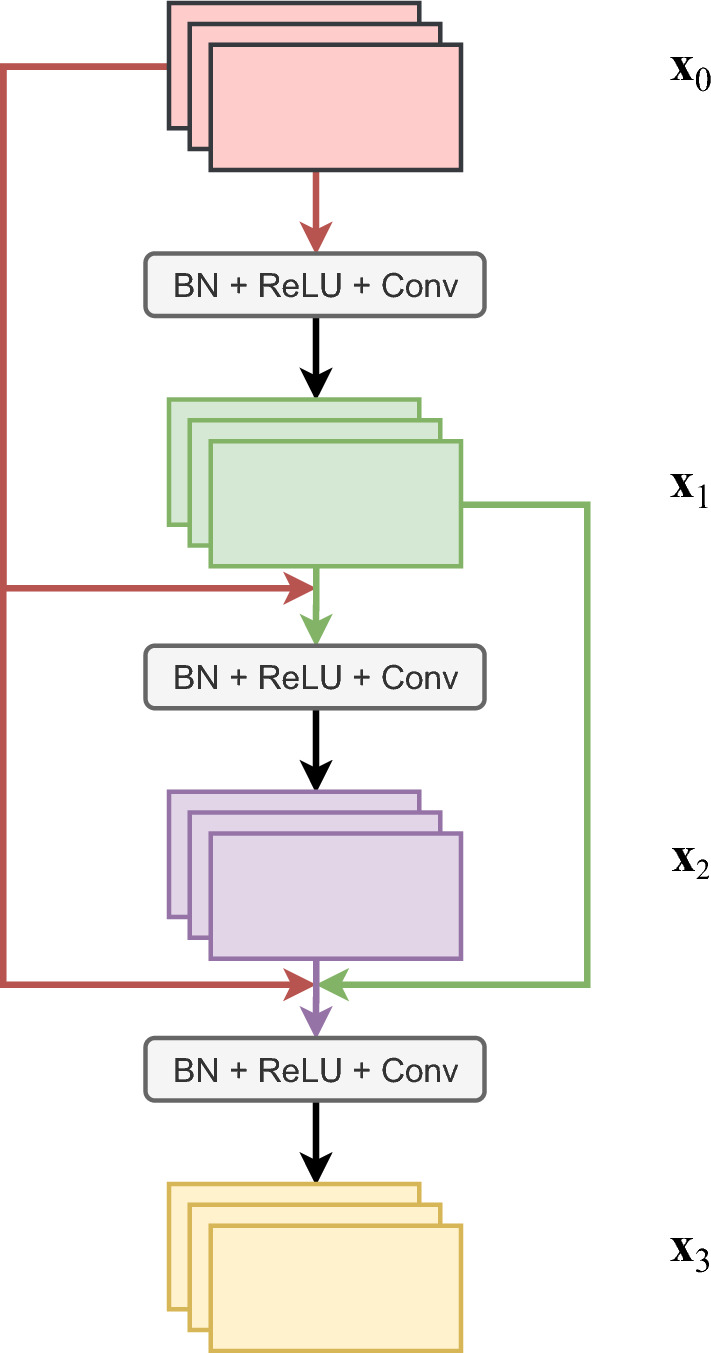
A pictorial representation of the dense connections (colored edges) in the DenseNet architecture. Modified from [33]

The DenseNet [33] architecture is also similar to the ResNet architecture. While ResNet adds skip connections between layers, DenseNet adds dense connections in-between layers. The output of a particular layer is directly connected to all subsequent layers of the network. This is highlighted in Fig. 3. The addition of these direct connections improves the parameter efficiency of the model while reducing redundancy at the same time. It also allows an improved flow of gradients through the network, similar to ResNet.

### Classifier combination methods

**Fig. 4:**
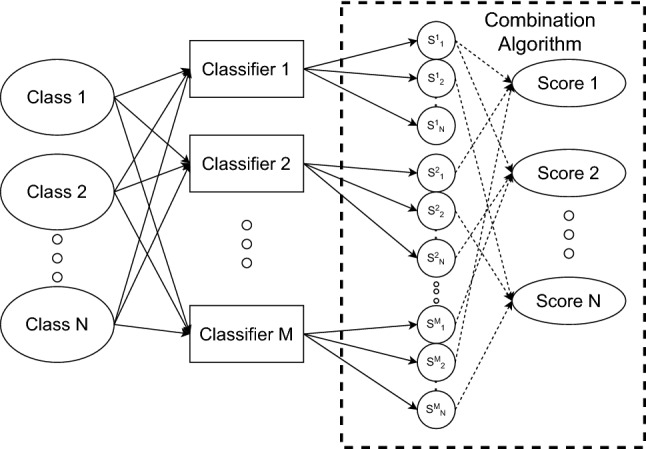
A pictorial description of the concept of the classifier combination in general. Modified from [34]

It is a popular approach to combine two or more classifier’s output using some combination or ensemble function to generate the combined output. The outputs of one single classifier can be represented as a vector where the dimension of the vector is the same as the total number of classes the classifier is trained to predict. So the problem of combination can be defined as to generate an N-dimensional vector from M such N-dimensional vectors (Fig. 4), where N is the total number of classes and M is the total number of classifiers and the ensemble function should minimize the amount of misclassification.

To build an ensemble function *f* in order to combine classifiers output we can consider two approaches. The first one is to take the outputs from the classifier and run some machine learning-based algorithm to generate the final output vector. So in other words the ensemble function works as a secondary classifier that takes the outputs of the primary classifiers as input. Dar-Shyang Lee proposed neural networks [35] to generate the combination output using the output vectors generated from individual classifiers. So in this way, *f* can be a neural network, support vector machine (SVM) or any other machine learning algorithm used for classification.

The other approach is to define *f* as simple functions such as sum, average or weighted average. In this way instead of learning from the outputs of the classifiers, it considers the output score or even the output prediction (argmax of the output vector) to generate the combined output vector.

The ensemble function *f* can operate on any level of the classifiers. So instead of using classifiers as class predictors we can use them as a feature extractor and execute *f* on this level. This concept makes more sense when learning algorithms are used as the ensemble function. Now different classifiers might learn some features better than other ones and *f* as the secondary classifier can learn those features. The ensemble function can also operate on output or confidence score level where it is not required to pass any architectural or feature-based knowledge of the classifiers to *f*. Score level combination is used popularly because it allows the combination of classifiers of different architectures.

#### Majority voting

This is a straightforward voting method that only considers the predicted classes of the classifiers and chooses the most frequent class label as the final output from the whole output set. One major drawback of this voting may result in a tie. Though Ho et al [9] discuss tie-breaking methods, generally the number of classifiers are taken as odd while using this method.

#### Sum rule (Soft voting)

Let us consider output of some $i^{th}$ classifier ($i \in [0,k]$) is $o_i = [s_i^0,s_i^1,...,s_i^C]$ where $s_i^j$ is the confidence score of $j^{th}$ class ($j \in [0,C]$). Now define majority as summation of the vectors ${s'}_i$ where ${s'}_i^k$ is 1 if only $argmax_j {s_i^j} = k$, any other value is 0. So if the final output vector $Y = [Y_0,Y_1,...,Y_C]$ is produced by majority voting then

1 $$\begin{aligned} Y_j = \sum _{i=0}^k {s'}_i^j \end{aligned}$$

We can simply use the concept of summation with only using $s_i^j$ by doing

2 $$\begin{aligned} Y_j = \sum _{i=0}^k s_i^j \end{aligned}$$

This method is also known as soft voting as we include the concept of voting but instead of only considering predictions, the confidence score is considered. We can further perform average or some normalization on the output values.

#### Borda count

This method is a voting technique that works on the rank level of the classifiers [37]. The confidence score $s_i^j$ in each classifier’s output is assigned with a rank $r_i^j$ in such a way that the highest score value gets the lowest rank value. In Borda count, the rank values are added to get the combined rank output $R = [R_0,R_1,...,R_C]$.

3 $$\begin{aligned} R_j = \sum _{i=0}^k r_i^j \end{aligned}$$

The final class is predicted by performing *argmin* on *R*. To enhance the method a weight value $w_i$ is attached to each classifier, which can be calculation by logistic regression [38] and the final rank is counted by taking the weighted sum.

**Table 1:** Example of the proposed ensemble method in comparison with sum-rule method (with average)

Classifier	Accuracy	Output	Calculated Weight	Weighted output
1	0.91	[0.97,0.03,0.0]	4.331	[4.201,0.129,0.0]
2	0.95	[0.22,0.55,0.23]	3.165	[0.696,1.741,0.728]
3	0.95	[0.0,0.72,0.28]	3.659	[0.0,2.635,1.025]
Weighted average		[1.633,1.502,0.584]		
Normalized score		[0.449,0.394,0.157]		
Predicted class		class-0		
Predicted class with sum-rule		class-1		

The weights are calculated using Eqs. 6 and 7. Then the weighted average is calculated using Eq. 8. The normalized scores the softmax output of weighted average. When the combination of the output scores is done by just averaging the score values, we get final output close to [0.39,0.43,0.18] denoting class-1 to be the final prediction, while the proposed method considers the confidence scores shown by the output of classifier-1 and reflects it in the final prediction

### Proposed method: inverted bell curve weighted ensemble

This section presents the mathematical formulation of the proposed ensemble methods. Let there be *C* classes in dataset and *k* classifiers trained on the dataset. In this paper value of *k* is taken is 3 but *k* can take any finite value. Let $s_i^j$ be the confidence score for $j^{th}$ class predicted by the $i^{th}$ classifier. The confidence scores are the output of softmax; hence, the output of some $i^{th}$ classifier will follow:

4 $$\begin{aligned} \sum _{j=0}^C s_i^j = 1 \quad \quad {where}\quad s_i^j \in [0,1] \end{aligned}$$

Now weight is assigned to each of the classifiers output using inverted bell curve function which is a function in form of

5 $$\begin{aligned} f(x) = \frac{1}{a} exp\left(\frac{(x-b)^2}{2c^2}\right) \end{aligned}$$

**Fig. 5:**
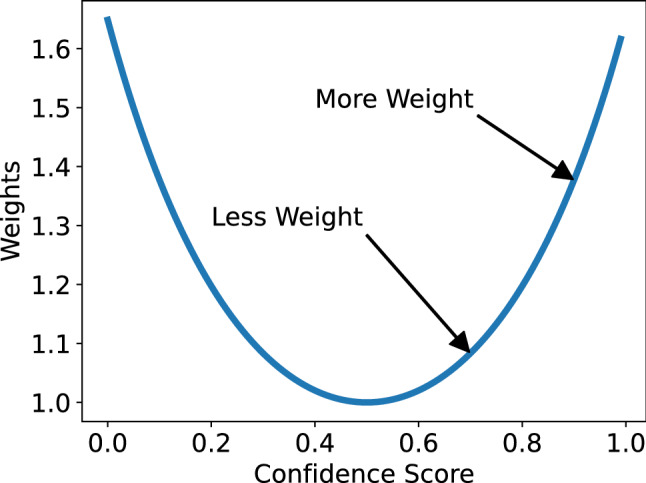
The plot of the function *f*(*x*) from Eq. 7 Here it can be observed that we have higher value of the weight as we approach both 1 and 0. So more weight is assigned to an output of a classifier when it not only classifies the correct class with highest confidence but also shows confidence that the sample data does not belong to the incorrect class with lower *s* value

The function *f*(*x*) is also known as the inverted bell curve (see Fig. 5). The inverted bell shape is particularly useful to implement this weighted averaging scheme. It can be observed that the shape of *f*(*x*) is more round at the bottom than any equivalent parabolic curve. We hypothesize that this helps in penalizing a wider range of low confidence score values, resulting in a better ensemble.

The parameter *a* is inversely proportional to the depth of the inverted bell. The value of *a* gets closer to 0 the bottom of the curve comes nearer to the *x*-axis. The parameter *b* controls the position of the centre of the curve bottom. At $x=b$, we can achieve the minimum value of *f*(*x*) where $a > 0$. The parameter *c* determines the width of the bell.

Let us consider the point $x=b$, where *f*(*x*) has its minima given $a > 0$, so as *x* is incremented or decremented we will get higher values of *f*(*x*), similar amount at both direction due to the fact that $(x-b)$ term is squared in the equation. This very idea is used in the context of assigning weights to the outputs of each classifier.

Let us consider two independent classifiers *P* and *Q* produce [0.8, 0.1, 0.1] and [0.5, 0.3, 0.2] as output confidence scores for some input *X*. Though both of these classifiers predict the *X* belongs to class-0, the classifier *P* does it more confidently. Therefore, while doing the weighted average of these scores, we must assign more weight to the classifier *P* for this output. In doing so, the property of *f*(*x*) discussed above is used. Let the minima of *f*(*x*) be at $x=0.5$, then we will get higher values of *f*(*x*) as we get closer to 0 or 1 because these are respectively lower and upper bounds for $s_i^j$. It can be easily shown that minima of *f*(*x*) exists at $x=b$. So the value of *b* is taken as 0.5 to satisfy our requirement. The value of *c* determines the range of the weights and it is chosen as 0.5 experimentally.

There may arrive a situation when some classifiers having very poor performance metrics over a dataset but for some instances it produces the outputs confidently. Therefore, without suppressing the classifiers’ impacts completely, we aim to weaken its contribution in the ensemble output, and we consider the accuracy of the classifier by taking $a=1/A_i,$ in *f*(*x*), where $A_i$ is the accuracy for the $i^{th}$ classifier. So the weight $w_i$ assigned to the output of $i^{th}$ classifier is

6 $$\begin{aligned}&w_i = A_i\cdot \sum _{j=0}^C f(s_i^j) \quad where \end{aligned}$$

7 $$\begin{aligned}&f(x) = exp(\frac{(x - 0.5)^2}{0.5}) \end{aligned}$$

The final output $[Y_0, Y_1, ..., Y_C]$ is generated by taking the weighted average of confidence scores across *k* classifiers using $w_i$, where

8 $$\begin{aligned} Y_j = \frac{1}{k}\cdot \sum _{i=0}^k w_i\cdot s_i^j \end{aligned}$$

We can further apply softmax on the calculated *Y* to normalize the output scores and obtain the final class probability. Finally, the class $\zeta$ is predicted from this output as

9 $$\begin{aligned} \zeta = {arg max}_{j} \{Y_j\} \end{aligned}$$

Table 1 shows an example of the proposed ensemble method where we take $C = 3$ and $k = 3$ and calculate with output confidences of these classifier.

## Results and discussion

### Dataset used

The proposed method is evaluated on two publicly accessible datasets of CXR images:

1. COVID-19 Radiography Database [10] - This dataset is comprised of 1,341 Normal, 1,345 Viral Pneumonia and 219 COVID-19 positive CXR images. This is also known as the Kaggle dataset.
2. IEEE COVID Chest X-ray Dataset [11] - This dataset contains 563 COVID-19 positive CXRs and 283 CXRs which are not diagnosed as COVID-19. As this dataset size is very small, image rotation is applied as a data augmentation technique to avoid over-fitting during model training. This dataset is also known as the Cohen dataset.

### Performance metrics

In this section, we first highlight the performance metrics that are used in the present work. Before defining the metrics, we define true positives, true negatives, false positives and false negatives in the context of classification. Thereafter, we mention the metrics.

The number of true positives *TP* denotes the number of items belonging to a particular class that are correctly predicted as belonging to that class.

The number of true negatives *TN* denotes the number of items not belonging to a particular class that are correctly predicted as not belonging to that class.

The number of false positives *FP* denotes the number of items not belonging to a particular class that are incorrectly predicted as belonging to that class.

The number of false negatives *FN* denotes the number of items belonging to a particular class that are incorrectly predicted as not belonging to that class.

Accuracy represents the fraction of labels that the model predicts correctly. It can be represented mathematically by Eq. 10. Oftentimes, it is represented in the percentage form.

10 $$\begin{aligned} Accuracy = \frac{TP + TN}{TP + TN + FP + FN} \end{aligned}$$

Precision is the ratio of the number of items correctly predicted as belonging to a class to the total number of items predicted as belonging to that same class. It can be mathematically represented by Eq. 11.

11 $$\begin{aligned} P = \frac{TP}{TP + FP} \end{aligned}$$

Recall is the ratio of the number of items correctly predicted as belonging to a class to the total number of items belonging to that same class. It can be mathematically represented by Eq. 12.

12 $$\begin{aligned} R = \frac{TP}{TP + FN} \end{aligned}$$

F1 score is the harmonic mean of precision and recall. It can be mathematically represented by Eq. 13.

13 $$\begin{aligned} F_1 = 2 \frac{P \times R}{P + R} \end{aligned}$$

A receiver operating characteristics (ROC) curve is a graphical plot that highlights the performance of a classifier at different thresholds. It is created by plotting the *TP* rate against the *FP* rate. The area under the curve (AUC) provides a metric for judging the performance of a classifier. The AUC value lies in the range [0.5, 1] with a value of 0.5 denoting the performance of a random classifier and a value of 1 representing a perfect classifier. Hence, the higher the AUC, the better is the classifier’s performance.

### Experimental results

**Fig. 6:**
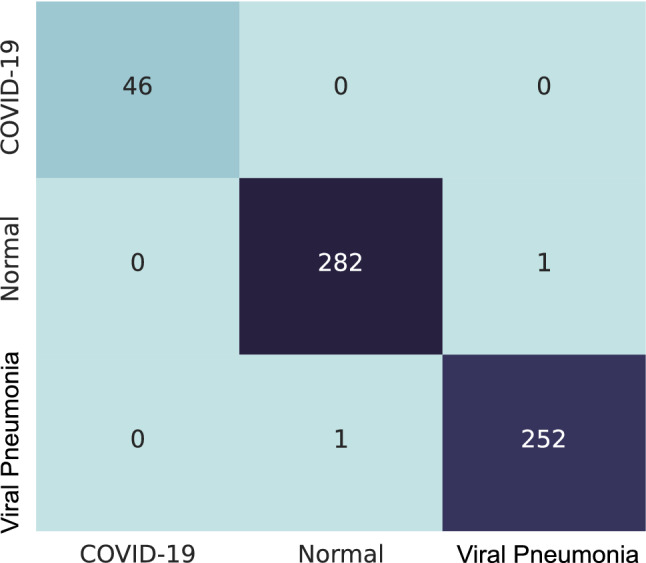
Confusion matrix for COVID-19 Radiography Database

The CXR images from the COVID-19 Radiography Database are trained on pretrained Denenet-161, ResNet-18 and VGG-16 separately with no frozen layers. This is a multi-class classification with the classes: ‘COVID-19’, ‘Normal’, and, ‘Viral Pneumonia’. The validation split used in this training is 0.2. The models are trained up to 100% training accuracy and no overfitting has been observed. The model accuracy, F1 score and AUC ROC calculated on the test set are shown in Table 2. The computation times of the transfer learning models are reported in Table 3. The proposed ensemble method is applied with the confidence score obtained from the trained classifiers and the metrics calculated based on the output of the ensemble are also shown in Table 2. Figure 6 shows the confusion matrix for this dataset on the test set. It is clearly observed that the proposed ensemble method has increased the accuracy significantly.

**Table 2:** Evaluation metric on COVID-19 Radiography Database

Model	Accuracy(%)	F1 score(%)	AUC(%)
DenseNet-161	98.97	99.25	99.63
ResNet-18	98.11	98.02	99.07
VGG-16	98.11	98.02	99.07
Proposed	99.66	99.75	99.99

**Table 3:** Computation time to train each individual transfer learning models

Model	Epochs	Time
DenseNet-161	20	45m 44s
ResNet-18	20	33m 46s
VGG-16	20	34m 13s

**Fig. 7:**
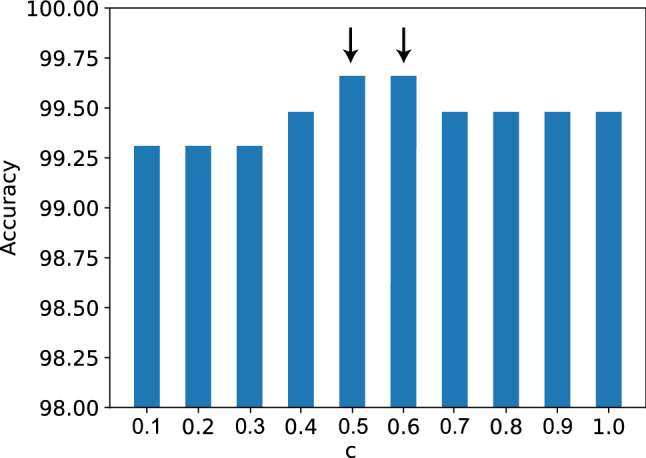
Effect of weight function parameter (*c*) on accuracy(in %) of the model. The arrows indicate the maximum accuracy obtained by the model

To determine the value of the parameter *c* in the Eq. 5, we have tested with multiple values of $c > 0$. Figure 7 shows the ensemble accuracy achieved for different values of *c* on the COVID-19 Radiography Database. We chose to proceed with the value $c = 0.5$ as it achieves the highest accuracy.

**Fig. 8:**
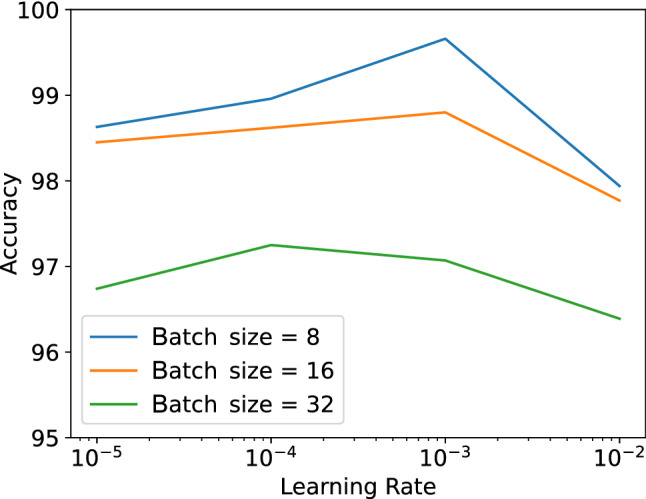
Effect of learning rate on the model’s accuracy for different batch sizes

For hyperparameter optimization, we use the grid search method. Figure 8 shows the accuracy score on the Radiography Database dataset for different values of batch size and learning rate. The accuracy shown in the figure is the proposed ensemble accuracy on the test set. When the learning rate is large, the model arrives on a sub-optimal final set of weights and due to large step size arriving on further optimal stages is not possible. With a small learning rate, there is always a possibility of reaching a locally optimal set of weights instead of the global one. So we observe worse performance for higher and lower values of learning rate. We can also observe that a smaller batch size produces better results due to the small size of the dataset used in this study.

For the experimentation, multiple ImageNet pretrained models are trained and tested on the CXR images and we have also tried all possible combinations of the models using the proposed ensemble method. Table 4 shows the Accuracy and F1 Score obtained on the said databases for the models and some of their combinations. Though the table clearly shows that the proposed ensemble method gives better performance than individual models for all of the combinations considered, we have decided to continue with DenseNet-161, VGG-16 and ResNet-18 as their combination produces the best result.

**Fig. 9:**
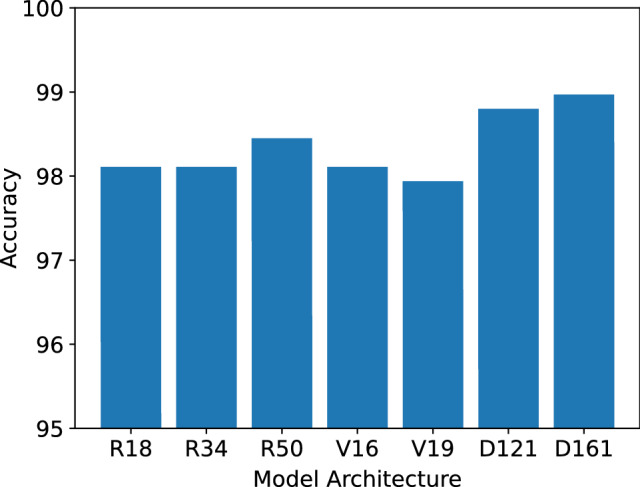
Pretrained Model Architecture vs Test Accuracy(in %). Only the initial letter and the numeric part of the architecture have been used to shorten the name. R, V and D stands for ResNet, VGG and DenseNet, respectively, as such, R50 denotes ResNet50

All the available architectures of the pretrained models have been taken under consideration for the experiment purpose. We choose the architectures that produce good results with low training time. Figure 9 shows the test accuracy on COVID-19 Radiography Database for different model architectures. Shortened names of model architectures are used in the mentioned figure such as R34 for ResNet34, V16 for VGG16 etc. An increment in the depth of a CNN model does not always guarantee better performance, we can observe that in the figure where VGG16 outperforms VGG19 by a tiny margin but DenseNet 161 outperforms DenseNet121. For ResNet architectures, we can observe that ResNet50 produces better accuracy than ResNet18 and ResNet34, but the time required to train ResNet50 is much higher than the other two. Though ResNet34 and ResNet18 perform similarly on this dataset, we continue with ResNet18 due to its faster training time.

**Fig. 10:**
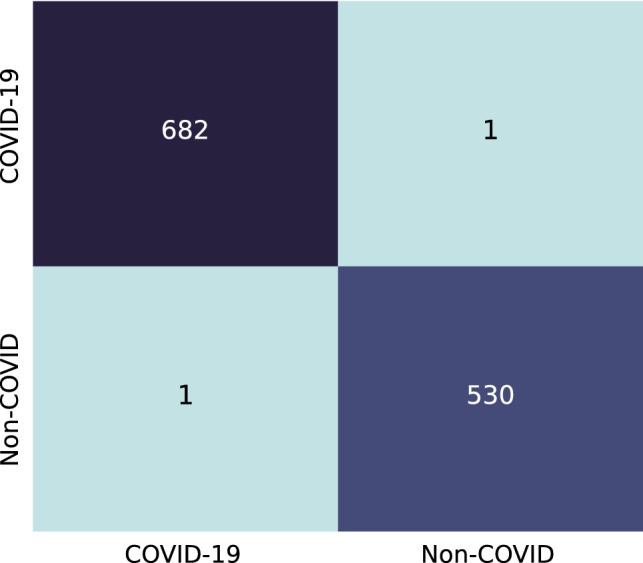
Confusion matrix for The IEEE COVID Chest X-ray Dataset

**Table 4:** Performance metrics for various pretrained model on COVID-19 radiography database

Models	Accuracy(in %)	F1 score
DenseNet-161 (1)	98.97	99.25
ResNet-18 (2)	98.11	98.02
VGG-16 (3)	98.11	98.02
Alexnet (4)	97.94	97.75
Inception-v3 (5)	98.80	98.78
1+3+4	99.31	99.26
1+2+4	99.31	99.26
**1+2+3**	**99.66**	**99.75**
2+3+5	99.49	99.34
1+2+3+5	99.49	99.34
1+2+3+4	99.31	99.26
1+2+3+4+5	99.49	99.34

Bold text suggests the best performance obtained within the table. Ensemble combinations are denoted by the model numbers concatenated with ‘+’ signs

The IEEE COVID Chest X-ray Dataset is similarly trained on the previously mentioned pretrained models. This is a two-class dataset with ‘COVID’ and ‘Non-COVID’ as classes. Table 5 shows the accuracy, F1 score and AUC ROC calculated from the trained models and ensemble of these three. Figure 10 shows the confusion matrix for this dataset.

**Table 5:** Performance evaluation (in %) on IEEE COVID Chest X-ray Dataset

Model	Accuracy	F1 score	AUC
DenseNet-161	99.59	99.51	99.57
ResNet-18	99.59	99.51	99.57
VGG-16	99.75	99.71	99.79
Proposed	99.84	99.81	99.99

**Fig. 11:**
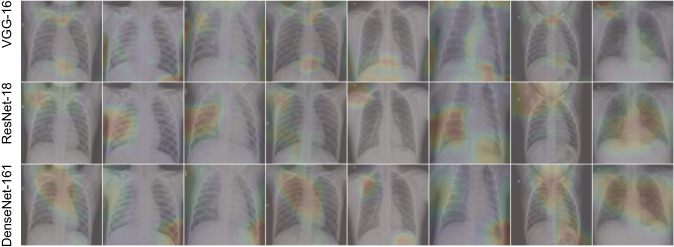
Grad-CAM performed on the last convolution layer for VGG-16 (top row), ResNet-18 (middle row) and DenseNet-161 (bottom row)

Table 6 shows the comparison between the popular ensemble techniques and the proposed method on of the datasets mentioned in this paper.

**Table 6:** Accuracy (in %) over different ensemble techniques

Ensemble methods	Radiology dataset	IEEE dataset
Borda count	98.11	99.59
Majority voting	98.97	99.59
Sum-rule	98.97	99.59
Proposed	99.66	99.84

We observe from Tables 2 and 5 that the present approach achieves the best results on both datasets. On the first dataset, the DenseNet-161 model produces the best performance and has a 99.66% accuracy. On the second dataset, the VGG-16 model achieves the best results and has an accuracy of 99.75%. However, the present ensembling approach outperforms both the above and achieves accuracy values of 99.66% and 99.84%, respectively, on the two datasets. This demonstrates the robustness of the model as compared to the three state-of-the-art CNNs considered for comparison.

In addition to the above, Table 6 also highlights the performance with some ensembling techniques that have been used in some recent works on COVID-19. As mentioned previously, these do not take into account the quality of the classifier predictions. It can be seen that the present approach also provides better results than all these ensembling techniques. This can be attributed to the fact that the present method favours the classifiers that predict classes with higher probabilities.

We also experiment on Kaggle Pneumonia Dataset to prove the robustness of the proposed method. This dataset contains 2530 Bacterial Pneumonia, 1345 Viral Pneumonia and 1341 Normal CXR images. Table 7 shows the test accuracy achieved with base models and proposed ensemble. It can be observed that the proposed method significantly increases the performance of the best accuracy. Hence, we can safely claim that the inverted Bell curve based ensemble method can be explored in future in other domains.

**Table 7:** Evaluation metric on Kaggle Pneumonia Dataset

Model	Accuracy(in %)
DenseNet-161	83.52
ResNet-18	83.14
VGG-16	83.71
Proposed	86.11

### Discussion

DL-based models like CNNs generally provide better performance than conventional white-box machine learning models techniques like regression, decision trees, etc. However, it is to be noted that DL-based models are black-box models in general. It is difficult to obtain explainability for the predictions which may be important in certain fields like medical image processing. Here, medical professionals want a prediction to come from the relevant artefacts present in the input image (X-Ray, CT scan, etc.) and not from irrelevant parts of the image like the background.

The work reported in [48] is one such work that provides an explainable machine learning approach for EEG-based brain-computer interface systems. In this work, the core prediction is performed using a CNN model. To introduce explainability in the system, the authors have used occlusion sensitivity analysis along with saliency maps segmentation through k-means clustering. Occlusion sensitivity analysis is a simple approach where patches of the input image are occluded using a mask and the effect on the output is observed. This is used to infer the regions of interest.

**Table 8:** Comparison of the proposed method with other deep learning methods in previous studies

Work ref.	Datasets	Accuracy (%)
Tang et al. [44]	COVIDx Dataset	95
Qiao et al. [45]	IEEE COVID CXR + Kaggle Pneumonia	79.67
Chowdhury et al. [47]	COVIDx	97
Turkoglu et al.[46]	COVID-19 Radiography Database + Kaggle COVID-19 + Kaggle Pneumonia	99.18
Proposed method	COVID-19 Radiography Database	99.66
	IEEE COVID CXR	99.84

The work in [49] is another work where the authors have introduced explainability in a machine learning framework. They have evaluated their approach for Glioma Cancer prediction and have shown a comparable performance with black box methods with the added advantage of explainable predictions. Besides, the work reported in [50] is a recent method, where an explainable deep learning framework has been presented for COVID-19 diagnosis in chest X-rays. The authors have utilized the Grad-CAM approach for obtaining explainability from their CNN base learners.

In the present work, we use Grad-CAM [39] to capture the region of attention for the three models used in this study. The idea behind Grad-CAM is to calculate the weighted average of the feature maps obtained from a particular layer in a model where the weights are the gradients of the feature maps calculated on the predicted class score. We choose the final convolution layer for Grad-CAM as it is considered to have the best compromise between detailed spatial information and high-level semantics [39]. In Fig. 11, the superimposed image of the Grad-CAM mask and the input CXR is shown. The rows in the image grid correspond to VGG-16, ResNet-18 and DenseNet-161, respectively, from top to bottom. Each column represents the same input CXR. The region of interest is shown as red spots in the figure. It can be observed that different models put attention on different regions of the same CXR which can prove to be useful for the combination of these models.

Figure 11 also shows that the regions of attention of the models are near the upper respiratory tract and alveolar lobes. The initial effects of SARS-CoV-2 are generally found in the upper respiratory tract. Further development of the virus results in fibrin accumulation on the alveolar region causing reduced gas exchange in the lung. The models individually pay attention to small and different regions looking for the textural changes caused by this fibrin accumulation [42]. So the proposed ensemble of these models can help in considering all of these textural changes found in the chest X-rays.

Table 8 compares the proposed method with some recent works in the same domain. Tang et al. [44] implement an ensemble of multiple snapshot models of COVIDNet [40] on the COVIDx Dataset. The authors use a weighted ageing ensemble technique to combine the snapshot models. Qiao et al. [45] use focal loss bases neural ensemble on a combined dataset of IEEE COVID Dataset and a Kaggle Pneumonia Dataset. Chowdhury et al. [47] use an ensemble of a number of EfficientNet snapshots on COVIDx dataset. Turkoglu et al. [46] apply Relief feature selection algorithm to select deep features from transfer learned AlexNet. This method is evaluated on a combined dataset made up of three public datasets.

**Table 9:** An example of a failure case

Model	Output score	Predicted label
ResNet-18	[0.2215, 0.5241, 0.2544]	1
VGG-16	[0.2293, 0.3107, 0.4600]	2
DenseNet-161	[0.2194, 0.5393, 0.2414]	1
Ensemble	[0.0434, 0.8095, 0.1471]	1
True Label	2	

It can be observed that ResNet-18 and DenseNet-161 yield wrong predictions. Though VGG-16 produces the correct label, it does so with very low confidence. The proposed method assigns less weight due to this low confidence score, producing the incorrect prediction

### Error case analysis

Table 9 shows a failure case encountered with the proposed method. Upon careful observation, it can be found that all of the predictions from the base learners are very weak. Not a single model has a confidence score over 60% for their predicted labels. Though the VGG network predicts the correct label, it has the lowest confidence score among all the other prediction confidences. Our method is unable to emphasize such low confidence score leading to incorrect prediction.

**Fig. 12:**
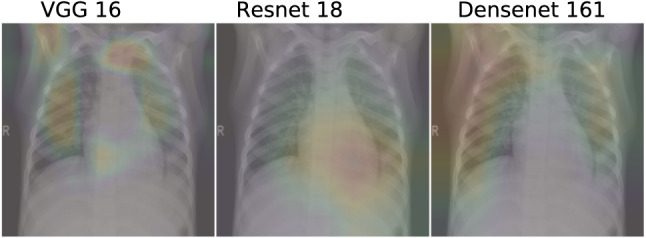
Grad-CAM results for the failure case mentioned in Table 9

Figure 12 shows the Grad-CAM results for a failure case. From the figure, it is noted that VGG-16 network mostly focuses on the lung regions as shown by the red regions in the heatmap. It produces the correct label prediction of 2 indicating viral pneumonia. However, if we consider the ResNet-18 network, it does not focus on the lungs for its prediction, which may explain the incorrect prediction of label 1 indicating a normal X-Ray. Finally, for the DenseNet-161 network, we see that it also focuses on the lung region. However, as compared to VGG-16, the focus is very dispersed as indicated by the reddish region in the top-left of the image. The incorrect prediction of label 1 can be attributed to this factor.

## Conclusion

In this work, we have developed an inverted-bell-curve-based ensemble of DL (or CNN) models for the detection of COVID-19 from CXR images. The concept of transfer learning is used to transfer and fine-tune the pretrained weights as the availability of COVID-19 CXR images are not abundant enough. We have used three such models to train on the available data and combined them at confidence score level using the proposed ensemble method which considers how confidently a classifier predicts the correct class with a high score value as well as identifies the wrong class as wrong with low score value.

The experimental results indicate that the combination of the CNN models using the proposed method produces better results than the individual models themselves. It is also notable that the proposed ensemble method gives superior results over existing confidence score level ensemble methods which do not consider the quality of the output of the classifiers.

## Limitations and future work

An obvious limitation of the present work is that the CNN classifiers may fail to detect the COVID-19 in CXRs of the patients in the early stages. This is because the CXRs may contain minor or no artefacts which the CNNs cannot detect as features. Hence, future works can focus on improving the feature extractors to combat the previous issue. To extract relevant features, recent techniques like hybrid supervised-unsupervised machine learning [41] can be used. Furthermore, recent architectures such as Vision Transformers [43] can be explored instead of CNNs. Preprocessing and postprocessing techniques can also be explored, especially those relevant for radiological images. In addition to the above, meta-heuristic algorithms can also be explored to improve the overall performance of the approach. Several recent works exist which have used meta-heuristics for hyperparameter tuning of the neural network to improve detection performance.

## Data Availability

The code can be accessed via the following GitHub repository: https://github.com/ashis0013/Inverted-Bell-Curve-Ensemble.
